# The Involvement of Photobiology in Contemporary Dentistry—A Narrative Review

**DOI:** 10.3390/ijms24043985

**Published:** 2023-02-16

**Authors:** Ionut Luchian, Dana Gabriela Budală, Elena-Raluca Baciu, Ramona Gabriela Ursu, Diana Diaconu-Popa, Oana Butnaru, Monica Tatarciuc

**Affiliations:** 1 Department of Periodontology, Faculty of Dental Medicine, Grigore T. Popa University of Medicine and Pharmacy, 700115 Iași, Romania; 2 Department of Prosthodontics, Faculty of Dental Medicine, Grigore T. Popa University of Medicine and Pharmacy, 700115 Iași, Romania; 3 Department of Dental Materials, Faculty of Dental Medicine, Grigore T. Popa University of Medicine and Pharmacy, 700115 Iași, Romania; 4 Department of Preventive Medicine and Interdisciplinarity (IX)—Microbiology, Faculty of Medicine, Grigore T. Popa University of Medicine and Pharmacy, 700115 Iaşi, Romania; 5 Department of Dental Technology, Faculty of Dental Medicine, Grigore T. Popa University of Medicine and Pharmacy, 700115 Iași, Romania; 6 Department of Biophysics, Faculty of Dental Medicine, Grigore T. Popa University of Medicine and Pharmacy, 700115 Iași, Romania

**Keywords:** phototherapy, laser, UV-A, UV-B, photostimulation, dentistry, PDT

## Abstract

Light is an emerging treatment approach that is being used to treat many diseases and conditions such as pain, inflammation, and wound healing. The light used in dental therapy generally lies in visible and invisible spectral regions. Despite many positive results in the treatment of different conditions, this therapy still faces some skepticism, which has prevented its widespread adoption in clinics. The main reason for this skepticism is the lack of comprehensive information about the molecular, cellular, and tissular mechanisms of action, which underpin the positive effects of phototherapy. However, there is currently promising evidence in support of the use of light therapy across a spectrum of oral hard and soft tissues, as well as in a variety of important dental subspecialties, such as endodontics, periodontics, orthodontics, and maxillofacial surgery. The merging of diagnostic and therapeutic light procedures is also seen as a promising area for future expansion. In the next decade, several light technologies are foreseen as becoming integral parts of modern dentistry practice.

## 1. Introduction

### 1.1. Concepts

While modern physics has succeeded in breaking down the components of nature into ever tinier and more exotic parts, light itself remains unchanged.

Healing with light, often known as heliotherapy or phototherapy, has been practiced for thousands of years [1,2]. Before the development of antibiotic medicines, increased sun exposure in sanatoria was a common therapy for infectious respiratory disorders such as tuberculosis (TB) [2].

However, one question remains: what is light, exactly? This brings us to a single aspect of light’s miraculous nature: it has no volume. Additionally, unlike negatively charged electrons, photons do not repel one another when packed into a tiny area. So, how many light angels can dance on the head of a pin? In theory, there is no limit.

Edgar Cayce and Rudolph Steiner were only two of the visionaries from the last century who foresaw the medical revolution that vibrational healing from color and light would bring [2]. Ever since, growth in the sector has been meteoric, and now light is employed in all kinds of areas, from laser eye surgery to telecommunications.

### 1.2. Historical Perspectives on Light Theories

As difficult as it is to comprehend light, it was far more challenging for the ancients. “Light is the activity of what is transparent,” Aristotle stated, quite cryptically [3]. This transparency was a vital quality of many substances; when triggered by the sun or fire, it generated light and color.

Empedocles, a fifth-century B.C. philosopher and poet, offered the great insight that light is a flowing material released by the sun and that we are unaware of its movement because it travels too quickly. Ancient Greek mathematicians, including Plato and Euclid, believed that the eyes emit some form of visual ray [2,4].

Alhazen’s idea of the camera obscura (in Latin “dark room”) used a small opening to project an inverted image of the outside world onto a wall. Leonardo da Vinci made the connection between the eye and the camera, a device invented centuries later.

Later, Descartes performed a somewhat spectacular inspection of an ox’s eyeball, scraping away the back of the eye and gazing into it. He noticed that the eye captures an inverted, upside-down representation of the world [2,5,6].

Light immediately flowed into Isaac Newton’s laboratory and was never the same again. Then, in the 1660s, Newton established that white light is composed of all the hues of the spectrum. He used a prism to break sunlight into a rainbow, then used another prism to cohere the colors back into white light.

One of the most important light-related discoveries was made by a Scot named James Clerk Maxwell in the 1860s. Maxwell had been investigating electricity and magnetism and discovered that they traveled through space at the speed of light. Light, he concluded, is an “electromagnetic” wave [2,7,8].

Clearly, light will continue to be highly useful in business, research, art, and our everyday, ordinary movements. Light pervades our experience at all levels of life. It is a wonderful instrument: a bearer of beauty and a source of life.

## 2. Spectrum of Light: From Visible to Invisible

Scientists create physical process models to better explain and predict behavior. The same is true for light energy. Photons simulate the particle-like characteristics of light. A photon has neither mass or charge. It transports electromagnetic energy and interacts with other discrete particles, such as electrons, atoms, and molecules [9].

Light (or similar radiations) is constituted of numerous 3D matter corpuscles moved by associated (electromagnetic wave-like) distortions in a universal medium.

Therefore, light exhibits all the characteristic properties of photons:

- The number of photons per unit time indicates the amplitude of light;
- The frequency of photons in a ray of light shows its intensity and color;
- The direction of spin of photons in a ray of light indicates its polarity.

A light source is represented by a continuous flow of photons, each of which has a certain energy that varies with its wavelength. The colors blue and red are only two examples of the spectrum of light’s wavelengths. Light waves are complex and carry light energy with them [9,10]. Light’s intensity, propagation direction, frequency or wavelength spectrum, and polarization are its most defining features.

When light travels through a medium, it interacts with that medium. The most important interactions are absorption and scattering. Absorption is a transfer of energy from the electromagnetic wave to the atoms or molecules of the medium, while scattering is the redirection of light caused by the light’s interaction with matter [10,11,12,13].

Light, or electromagnetic radiation (EMR), may be thought of as a continuous wave of light particles. These photons, or “light particles,” oscillate and spin as they travel through space [12,13,14].

**Figure 1:**
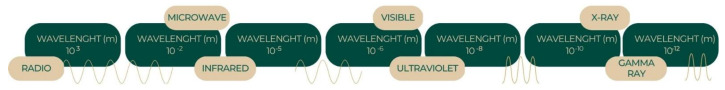
Light spectrum.

Generally, electromagnetic radiation (EMR) is classified by wavelength into radio waves, microwaves, infrared, the visible spectrum that we perceive as light, ultraviolet, X-rays, and gamma rays. Visible light is usually defined as having wavelengths in the range of 400–700 nanometers (nm) between the infrared (longer wavelengths) and the ultraviolet (with shorter wavelengths), known as UV, as seen in Figure 1 [15,16].

As a result of differences in vibration frequency and photodynamic reactivity, the effects of photons in various parts of the electromagnetic spectrum (EMS) on organic and inorganic materials are also quite different. Radiation with shorter wavelengths, such as gamma rays and X-rays, has a tendency to ionize materials; radiation with longer wavelengths, such as radio waves, is comparatively harmless [12,13,14].

## 3. Light in Modern Dentistry

### 3.1. Infrared Light and Near Infrared Light

While visible and ultraviolet light may only penetrate the skin and deeper layers of tissue by a few microns apiece, infrared radiation can travel 20–30 mm through a variety of tissues and may have far more profound effects, causing changes in circulating cytokines [17].

Dental tissue optics has been created as a noninvasive tool for early caries diagnosis as part of the ongoing quest for more precise diagnostic procedures. Novel and promising optical imaging uses near-infrared (NIR) light for the early detection of dental caries lesions and the evaluation of lesion severity [18]. The technique is noninvasive, does not include the use of ionizing radiation, and is thought to be more sensitive to early demineralization than dental radiography. 

The NIR (1310 nm) imagery was compared to radiography images by Jones et al. [19]. Poor radiographic contrast was seen for simulated approximal lesions in tooth pieces of various thicknesses. When the lesion was irradiated with near-infrared light, however, the line between the lesion and the healthy enamel around it stood out sharply.

Bühler et al. conducted an analysis in which imaging reflecting occlusal caries lesions was compared with radiographic imaging to evaluate the potential of an NIR laser in the identification of early occlusal caries lesions. NIR pictures were again shown to be superior to radiographic images, as was the case in the aforementioned investigation [20].

Compared to radiography, all of these investigations showed that the NIR TI approach was highly sensitive and specific. The method has the potential to enhance the regular monitoring of enamel lesions during preventative intervention, and it can be valuable for early caries diagnosis and patient follow-up [21].

### 3.2. High-Energy Visible (HEV) Light

High-energy visible (HEV) light, also known as “blue light,” is the term used to define light with a wavelength between 400 and 500 nm and lower energy than UV [22]. Due to blue light’s superior safety profile compared to UV light and its comparatively reduced photodegradation of the molecules it irradiates, it has attracted a great deal of interest as a potential therapy for a variety of conditions [23].

In vitro and in vivo studies have shown that blue light is deadly to bacteria and can kill both Gram-negative and Gram-positive microorganisms. The 402–420 nm spectral range is the most effective antibacterial spectral range; however, it has also been observed that the 455 nm and 470 nm wavelengths have antimicrobial potential against some bacterial species (e.g., *S. aureus*) [24]. Additionally, it has been determined that blue light can kill the anaerobic oral pathogens *Prevotella, Porphyromonas*, and *Fusobacterium* [25].

The absorption of electromagnetic energy in the blue light spectrum by electrons in molecular orbitals can cause photochemical processes or the internal conversion of light to heat. Since this is the case, it is plausible that the employment of light irradiation units in dental therapy warms the oral tissues. It is common knowledge that the mucous membranes of the lips are particularly vulnerable to the high temperatures produced at the irradiators’ tips [26]. A rubber dam will not prevent mucosal injury from occurring due to direct heat stimulation. As a result, during dental restoration treatments, the only place the light points should be placed is directly over the restoration. However, a common curing lighting with an output power of >600 mW/cm^2^ is currently utilized in clinical practice. 

As a safety measure, dentists should be aware that irradiation time during blue-light-based operations should be kept to a minimum. It is also important to monitor how far away the irradiation source is from the target. Some dentists believe that alternative strategies, such as keeping some space between the light’s tip and the tooth in question or dialing back the irradiance, can help to prevent heat injury to the pulp. Nevertheless, the curing of composite resin in low-light portions of the oral cavity, such as in the cervical regions of class II cavity preparations, might be compromised if the light tip’s radiant exitance values decrease as distance from the target tooth increases [27,28,29].

There has been an increase in the demand for tooth whitening as patients place greater emphasis on the aesthetics of their mouth and teeth, including the color of their teeth. One of the most common whitening techniques involves applying a solution containing 25–40% hydrogen peroxide to the teeth’s surfaces. Halogen curing lights, LEDs, diode lasers, argon lasers, and plasma arc lamps are just some of the blue-light-producing units used in tooth bleaching, which have been developed to improve the activation of hydrogen peroxide in a shorter amount of time and thus produce more desirable cosmetic results [30,31,32]. For this reason, blue light is crucial in contemporary dental care.

### 3.3. Ultraviolet Light

Extreme ultraviolet (below 100 nm), far ultraviolet (100–200 nm), middle ultraviolet (200–300 nm), and near ultraviolet (300–380 nm) are the four zones often used by physicists to investigate UV photons. However, three groups are commonly identified based on UV interactions with biological materials. The wavelengths of ultraviolet (UV) C light are between 100 and 280 nanometers, those of ultraviolet (UV) B light are between 280 and 315 nanometers, and those of ultraviolet (UV) A light are between 315 and 380 nanometers [33].

Dermal 7-dehydrocholesterol absorbs ultraviolet light between 290 and 315 nm, changing it into pre-vitamin D3, which then isomerizes into vitamin D3 and aids the immune system [34].

Ultraviolet radiation (405 nm) has several uses beyond just stimulating vitamin D production; it may also be used to disinfect or sterilize surfaces, air, and water, eradicating *Pseudomonas aeruginosa* in plumbing systems. Germs are killed by ultraviolet (UV) radiation because it damages DNA, forming thymine dimers that are difficult to repair [35].

Microorganisms are killed by ultraviolet light at far lower irradiation fluences than those caused by visible light. UV-C wavelengths about 260 nm are particularly effective for this purpose. UV light’s high photon energy means that it does not take much irradiance to create detrimental consequences, despite claims that the advantages of UV at low doses vastly exceed its adverse impact [36,37].

The oral cavity provides a favorable setting for the clinical use of ultraviolet UV irradiation technology for the management of polymicrobial biofilms in periodontal and peri-implant microbiomes [38,39]. However, endodontic infections and inflammation in root canals may be where it clearly emerges [40,41]. Infected root canals could potentially be treated with UV irradiation [42,43,44], and we believe that the application of narrow UV spectra will destroy microorganisms, stimulate tissues and cells, and cause the release of chemokines, cytokines, and biomarkers (CCBMs) that maintain periapical tissue health.

In 2019, Morio et al. analyzed the cytotoxic effects of 255 and 405 nm UV LED on human embryonic palatal mesenchyme (HEPM) cells and gingival fibroblasts in addition to evaluating the antimicrobial killing effects of 255 and 405 nm UV LED on *E. faecalis*. *E. faecalis* was considerably less likely to survive after being treated with 255 nm LED than 405 nm LED [44].

The trade-off between UV irradiation’s antibacterial effectiveness and its safe usage and detrimental effects on host tissues has long been a source of worry in both medical and dental contexts. Reed eloquently pointed out that UV irradiation’s potential to generate tissue and cell damage is strongly linked to the depth to which it can penetrate (which is determined by the wavelength) [45].

In contrast with UV-A and UV-B, UV-C is the most biologically active radiation, and it poses no risk to people. In contrast with the deeper penetration of UV-B and UV-A rays, UV-C rays are absorbed by the human skin’s outermost dead layer. The practical designs of ultraviolet germicidal irradiation fixtures are also improving, becoming more efficient while remaining safe, but new ideas are required to further increase efficiency and effectiveness while keeping production expenses low [46].

Researchers have been able to reevaluate the antimicrobial and cytotoxic properties of very specific wavelengths and spectra thanks to the availability of new UV LED, allowing them to pinpoint the wavelengths, powers, and doses of irradiation that maximize antimicrobial activity while minimizing cytotoxicity to tissue and cells. Reports reveal that optimum antibacterial activity takes place between 255 and 280 nm, whereas general antimicrobial activity occurs between 200 and 400 nm [47,48,49]. 

For instance, during the pandemic period, the American Dental Association (ADA), as well as most European dental organizations, recommended the use of UV lamps and other air purifiers and high-efficiency aspiration during treatments [50,51,52]. 

### 3.4. Shortest Wavelengths-X-ray

The wavelength of an X-ray is just 0.01–10 nm, much shorter than the wavelength of UV light. In the medical field, X-rays are primarily associated with imaging procedures including traditional X-rays, CBCT, and OPG. DNA damage and tissue destruction, which can be either pathological or therapeutic, are more likely to occur with shorter light beams [53].

X-rays have obviously made the transition from the world of unseen enigmatic energy to everyday conventional medical practice; depending on how they are employed, they can inflict significant damage or deliver great advantages [54].

## 4. Light-Sources in Dentistry Lasers

LASER is the abbreviation for Light Amplification via the Stimulated Emission of Radiation. Since Miaman first introduced the use of the laser in dentistry in the 1960s [55], researchers have been exploring its many potential clinical uses. Soft or cold lasers, based on semiconductor diode devices, are compact, low-cost devices used predominantly in applications, while hard lasers, such as carbon dioxide (CO_2_), neodymium yttrium aluminum garnet (Nd:YAG), and erbium doped yttrium aluminum garnet (Er: YAG), offer both hard tissue and soft tissue applications, but have limitations due to their high costs and their potential to induce thermal damage [55,56]

### 4.1. Mechanism of Action

Dental lasers transmit light to the tissue through an active medium, which can be a gas, a crystal, or a solid-state semiconductor. This is the primary factor in determining the laser’s wavelength (Table 1) and other features.

Laser-generated light energy can interact with tissue in four distinct ways [57,58]. All four of these processes: reflection, transmission, scattering, and absorption, are possible.

### 4.2. Types of Lasers

#### 4.2.1. Carbon Dioxide Laser (CO_2_)

Since the CO_2_ laser wavelength is highly selective for water, it may quickly and effectively remove soft tissue and stop bleeding with minimal penetration [59]. Soft tissue surgery is best performed using CO_2_ (10,600 nm), Nd:YAG (1064 nm), DL (800–980 nm), Er:YAG (2940 nm), or Er,Cr:YSGG (2780 nm) lasers. In addition to being a very conservative technique, its benefits include an increase in tissue temperature that aids in hemostasis and decreases microbial growth [60].

#### 4.2.2. Neodymium Yttrium Aluminum Garnet Laser (Nd:YAG)

This is a highly efficient surgical laser because the Nd:YAG wavelength is well absorbed by pigmented tissue. Research on the use of the Nd:YAG laser for nonsurgical sulcular debridement in periodontal disease management has been conducted in addition to its surgical applications [61].

#### 4.2.3. Erbium Lasers

Lasers of the erbium “family” come in two varieties, one with a longer wavelength (Er, Cr: YSGG; yttrium scandium gallium garnet) and the other with a shorter one (Er: YAG; yttrium aluminum garnet). Erbium lasers have the largest absorption of water and the strongest affinity for hydroxyapatite of any dental lasers. Because of this, they are the preferred laser for restoring dental hard tissues [62].

In vitro investigations have demonstrated that both Er:YAG and Er,Cr;YSGG lasers cause alterations in the surface morphology of treated roots, making them more irregular and rough [63].

#### 4.2.4. Diode Lasers

Diode lasers generate laser wavelengths in the 810 nm to 980 nm range using a solid-state semiconductor active medium composed of aluminum, gallium, arsenide, and sometimes indium.

Aesthetic gingival re-contouring, soft tissue crown lengthening, soft tissue impacted tooth exposure, inflamed/hypertrophic tissue removal, frenectomies, and photostimulation of aphthous/herpetic lesions are only a few of the operations that fall under this category [64].

For noninvasive periodontal therapy, dentists can employ a combination of diode lasers (DL) (808–904 nm) and neodymium-doped yttrium–aluminum–garnet (Nd:YAG; 1064 nm), erbium-doped yttrium–aluminum–garnet (Er:YAG; 2940 nm), and erbium-chromium [57,65].

Sulcular debridement (the removal of the sulcular epithelium from the periodontal pocket) and the promotion of the decrease in supra- or subgingival periodonto pathogenic bacteria are both indications for the use of the Nd:YAG laser and the DL, respectively [66].

The use of lasers in hard tissue applications and soft tissue surgeries has progressed to a highly refined stage over the course of several decades, and additional refinement is possible. Laser-based photochemical reactions’ ability to zero in on individual cells, pathogens, or chemicals has also been highlighted for potential use in the future.

Taking into account the classification of Placek and according to recent literature, it seems that the diode laser surgery techniques are superior in terms of haemostasis, surgical time, pain, edema, post-surgical inflammation, and healing time when compared with conventional surgery [67].

## 5. Dental Therapeutic Strategies

### 5.1. Photodynamic Therapy

Unlike other methods of tissue and cell death, photodynamic treatment (PDT) is both noninvasive and extremely specific. It relies on the fact that a photosensitizer (PS), molecular oxygen, and visible or near-infrared (NIR) light must all be present simultaneously, although none of these things are lethal or harmful to cells or tissues on their own. In an ideal scenario, the PS is absorbed and stored mostly in the intended cells [68,69]. 

### 5.2. Mechanism of the Photodynamic Action

**Figure 2:**
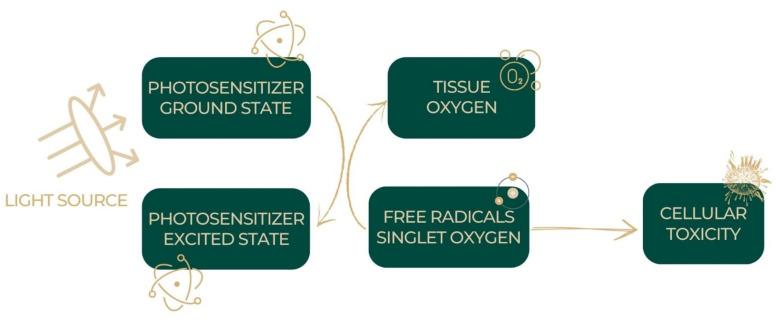
PDT mechanism.

The photodynamic response can occur via two major processes. Both rely heavily on oxygen molecules within living cells. Both methods have the same initial phase. After entering the cell, a photosensitizer absorbs photons at a certain wavelength that corresponds to its absorption spectrum (AS), causing it to transition from its ground (singlet) to its excited (singlet) energy state (S° to S1). While some of the energy is lost as fluorescence, the rest is used to excite a photosensitizer molecule into its active triplet state, T1. (Figure 2).

### 5.3. PDT in Dentistry

According to the scientific literature, PDT uses can be divided into several groups (Table 2) [70]. 

Xu et al. found that a 5 min exposure to 665 nm laser light at 20 and 40 mW/cm^2^ was sufficient to kill endodontic bacteria in vitro. Gingival fibroblasts and osteoblasts showed no apoptotic changes after laser therapy, demonstrating the safety and efficacy of PDT [77].

PDT (665 nm, 60 J/cm^2^) applied in vitro reduced the lifespan of *E. faecalis* in dental root canals by 77.5% [78]. A five-log decrease in microbial growth was seen in recent research, which used a combination treatment of root canal surgery and PDT (660 nm, 15 J). In contrast with conventional endodontic surgery, this approach yielded better results. Furthermore, after 36 months of therapy, the periapical infection area decreased by 78% [79].

Numerous studies have demonstrated that PDT is an effective supplement to scaling and root planning for the treatment of aggressive periodontitis [80]. Adolescents are more prone to developing a localized type of aggressive periodontitis, a rare illness. The subgingival microbiota of young people with periodontitis contains a greater concentration of actinomycetemcomitans according to research by Zambon and colleagues [81].

Park et al. [82] demonstrated the feasibility of toluidine blue O (TBO)-mediated PDT as a noninvasive supplementary method for the treatment of periodontitis. Many bacteria, including certain oral microbes, can be inactivated by PDT, according to some in vitro investigations [83,84,85,86]. *Porphyromonas gingivalis, Aggregatibacter actinomycetemcomitans, Fusobacterium nucleatum, Prevotella intermedia*, and *Streptococcus sanguis*, all of which have been linked to periodontal disease, were demonstrated to be strongly inhibited by aPDT under a variety of settings [87].

Park et al. [88] found that PDT was selective against periodontitis pathogenic bacterial while having no effect on resident oral bacterial growth. Moreover, its cytotoxic impact on normal periodontal cells was shown to be below the level typically seen in antiseptics.

For localized infections, the therapeutic use of PDT is thought to cause minimal harm, as confirmed by Hu et al. [89]. These results are consistent with PDT being the mechanism responsible for killing off microorganisms. When a certain wavelength of laser interacts with a photosensitizer dye, the dye molecule is excited and oxidized to generate singlet oxygen, leading to a reduction in the total number of bacteria [90].

Antifungals, both topical and oral, are commonly prescribed to treat stomatitis. However, this disease is common because of antifungal drug resistance, and it tends to reoccur. Prolonged and frequent usage increases the risk of strain resistance to pharmacological treatments and unwanted consequences [91,92]. Denture stomatitis can be a painful condition; however, antimicrobial photodynamic treatment (PDT) has been identified as a potential solution [93]. 

The use of antimicrobial PDT is desirable because it avoids the need for standard medication therapy, shortens the duration of treatment, and prevents the need for extended drug intake or excessive drug dose [94,95]. Using phototherapy in conjunction with nystatin was designed to drastically lower colony numbers, as opposed to only using the antifungal agent. Because of the alterations induced by PDT, nystatin is better able to enter fungal cells and bind to the ergosterol in their membranes, resulting in cell destruction and necrosis [96].

Always on the lookout for new ways to improve the accuracy of chemo-mechanical root canal cleaning, endodontists have developed several adjunctive techniques. As a result, PDT methods were offered as an alternative method to standard root canal endodontic preparation. This is carried out with the goal of eliminating as many germs as possible from the root canal structures [97,98]. Many variables have been linked to its success.

Considering this, photodynamic treatment is gaining favor not just with doctors, but with patients as well. The rapid progress and advancements in technology and optoelectronic equipment might promote the use of the photodynamic approach in clinical care. Although antibiotics are often used to treat periodontitis and peri-implantitis, PDT appears to be a safer alternative.

### 5.4. Photobiomodulation (PBM)

Photobiomodulation, also known as low-level light therapy (LLLT), has been attracting attention in the dental field due to its potential to improve a wide range of oral conditions. Due to its anti-inflammatory, antinociceptive, and antibacterial properties, photobiomodulation therapy (PBMT) has emerged as a potential and successful therapeutic option for the management of oral mucositis, periodontology, temporomandibular disorder (TMD) pain, dental implant osseointegration, and orthodontic tooth movement, which has been investigated in several studies [99,100,101].

In the systematic review, Zadik et al. [102], studied the effectiveness of LLLT in the management of oral mucositis and associated pain in cancer patients, which is a frequent side effect of chemotherapy and radiation treatment. According to the study, using specific LLLT settings can be recommended for the prevention of oral mucositis.

Injured nerves produce inflammatory mediators of the arachidonic acid family; however, low-level laser treatment has been found to decrease this production while also stimulating neuronal maturation and regeneration. [103,104].

Theodoro et al. [57,63] examined the use of LLLT in the treatment of periodontal disease. LLLT combined with non-surgical periodontal therapy was found to be helpful in lowering inflammation, accelerating bone and gingival tissue regeneration, and reducing periodontal surgery postoperative discomfort.

Several studies have shown that PBM therapy effectively reduced pain and improved outcomes in patients with temporomandibular disfunction (TMD) and dental implant osseointegration [105].

According to Zheng et al. [106], LLLT therapy can effectively accelerate orthodontic tooth movement and minimize the discomfort associated with traditional orthodontic treatment.

A recent systematic review has indicated that utilizing LLLT can be an effective method to decrease tooth sensitivity after dental bleaching [107].

### 5.5. Photodynamic Inactivation

One of the most well-known light-based treatments used to kill bacteria and other microbes is called photodynamic inactivation. 

Microorganisms are susceptible to photo-inactivation if three conditions are met: oxygen, a photosensitizer that can convert light energy into some harmful downstream product(s), and light of the proper wavelength matched with the absorption spectrum of the photosensitizer.

Every photosensitizing dye used in PDI, whether methylene blue, Rose Bengal, hypericin, xanthenes, etc., has a slightly different molecular conformation, and each is paired with a certain wavelength that generates a significant enough amount of reactive oxygen species (ROS) to kill the target microorganism [108].

As early as the 1920s, Schultz and Krueger used light waves and methylene blue to kill *Staphylococcus* bacteria, demonstrating the principle of photodynamic inactivation of microorganisms [109].

Despite these early accomplishments, therapeutic interest in photodynamic inactivation of bacteria and viruses has recently emerged in the last 50 years, as research in the subject has been substantially advanced by technological discoveries that have given rise to more efficient light sources.

## 6. Conclusions and Perspectives

Although mainstream medicine has traditionally prioritized pharmacological and surgical treatment methods, full-spectrum light therapy has emerged as a credible alternative therapeutic option and is now being used in a variety of settings.

In fact, there is currently promising evidence for the use of light therapy across a spectrum of oral hard and soft tissues and in a variety of important dental subspecialties, such as endodontics, periodontics, orthodontics, and maxillofacial surgery.

The merging of diagnostic and therapeutic light procedures is also seen as a promising area of future development. In the next decade, several light technologies are predicted to become integral components of the practice of modern dentistry.

To conclude, photobiology plays a crucial role in changing paradigms regarding optimizing or implementing new clinical protocols in contemporary dental medicine.

## Figures and Tables

**Table 1:** Types of lasers used in dental medicine and their wavelength.

	Lasers Used in Dentistry								
	Argon Laser	Potassium-Titanyl-Phosphate (KTP) Laser	Helium-Neon Laser	Low-Level Laser (LLL)	Diode Laser (DL)	Nd: YAG Laser	Er,Cr: YSGG Laser	Er:YAG Laser	CO_2_ Laser
**Wavelength [nm]**	488, 515	532	633	632–904	635, 670, 810, 830, 980	1064	2780	2940	9300, 10,600

**Table 2:** The role of PDT in dentistry.

Authors	PDT Uses Groups
Warrier et al., 2021 [71]	Photodynamic antimicrobial therapy (aPDT)
Takasaki et al., 2009 [72]	Photodynamic antimicrobial chemotherapy (PACT)
Rossoni et al., 2010 [73]	Photodynamic disinfection (PDI)
O’Riordan et al., 2006 [74]	Antimicrobial photodynamic inactivation (aPDI)
Späth et al., 2014 [75]	Lethal photosensitization
Bergmans et al., 2008 [76]	Photoactivated disinfection (PAD)

## Data Availability

All data are available from corresponding authors upon reasonable request.
